# Effect of self-paced sprint interval training and low-volume HIIT on cardiorespiratory fitness: the role of heart rate and power output

**DOI:** 10.3389/fphys.2025.1484722

**Published:** 2025-02-05

**Authors:** Katie L. Hesketh, Sam O. Shepherd, Anton J. M. Wagenmakers, Matthew Cocks, Juliette A. Strauss

**Affiliations:** ^1^ School of Sport, Exercise and Rehabilitation Sciences, University of Birmingham, Birmingham, United Kingdom; ^2^ Research Institute for Sport and Exercise Science, Liverpool John Moores University, Liverpool, United Kingdom

**Keywords:** high-intensity interval training, cardiorespiratory fitness, self-paced, real-world, heart rate

## Abstract

**Aim:**

The primary aim was to assess the efficacy of self-paced sprint interval training (SIT) with low-volume high-intensity interval training (LV-HIIT) when performed without encouragement on improving cardiorespiratory fitness (CRF). A secondary aim was to explore whether the effort exerted during protocols [power output (PO) and heart rate (HR)] influenced the change in CRF.

**Methods:**

In a randomised cross-over design, 82 previously inactive adults (m/f: 26/56, 28 ± 10 years, BMI 25 ± 3 kg m^−2^) undertook 6-weeks of self-paced SIT (4–8 × 30 s with 120 s recovery) or LV-HIIT (6–10 × 1 min with 1 min recovery) separated by a 4-week washout period. Sessions were completed 3×/week using WattBikes, and a target of >80% HRmax was suggested during the intervals. Markers of cardio-metabolic health were assessed before and after each intervention.

**Results:**

Training increased VO_2peak_ (SIT +3.1 ± 0.4 mL kg^−1^ min^−1^, LV-HIIT +2.7 ± 1.2 mL kg^−1^ min^−1^, *P* < 0.001) and decreased body fat % (*P* = 0.002), aortic pulse wave velocity (*P* = 0.002) and glucose tolerance 120 min following an oral glucose tolerance test (*P* = 0.024), with no difference between protocols (*P* > 0.05). When grouping participants into tertiles based on HR and PO responses (n = 27), those achieving a low HR had similar changes in VO_2peak_ compared to the high HR group in both interventions (*P* > 0.05). For LV-HIIT, participants in the highest tertile for peak PO had a greater change in VO_2peak_ compared to all other participants (Low 1.8 ± 4.1 mL kg^−1^ min^−1^, Medium 1.9 ± 3.3 mL kg^−1^ min^−1^, High 4.3 ± 3.6 ml kg^−1^ min^−1^, *P* = 0.020).

**Discussion:**

Six-weeks of self-paced SIT and LV-HIIT induce comparable improvements in CRF, body composition, arterial stiffness and glucose tolerance. Importantly, higher HR did not elicit superior changes in CRF, but PO achieved during LV-HIIT may influence improvements.

## Introduction

High-intensity interval training (HIIT) has been proposed as a time efficient alternative to traditional moderate-intensity continuous training (MICT) (Gillen and Gibala, 2013). HIIT is an umbrella term which refers to physical exercise characterised by brief intermittent bursts of vigorous activity, interspersed by periods of rest or recovery (Gibala, 2007). HIIT can then be further sub-categorised based on factors such as interval duration and intensity. Protocols which contain <15 min of high intensity work per session, with a target interval intensity >80% HR_max_ are defined as low-volume HIIT (LV-HIIT) (Sabag et al., 2021). A form of low-volume HIIT is sprint interval training (SIT) which involves ‘all-out’ or supramaximal efforts (>100% maximal work rate) (Weston et al., 2014).

A recent meta-analysis reported similar improvements in cardiorespiratory fitness (CRF) following lab-based SIT and HIIT (de Oliveira-Nunes et al., 2021). However, the studies included were mostly conducted using specialised research equipment unavailable to the general population in the presence of researchers, who often provided constant encouragement. Therefore, little is known regarding the efficacy of SIT or LV-HIIT outside of a controlled environment. Furthermore, it has been stated that SIT is extremely demanding, and may not be tolerable for much of the sedentary population (Little et al., 2010). As such, Hardcastle et al. (2014) have argued that the transfer of SIT to the ‘real-world’, where inexperienced sedentary individuals are unlikely to have constant encouragement and in which responsibility is placed on them to self-select the appropriate exercise intensity, is likely to be problematic. In this context, self-paced high-intensity interval exercise (HIIT) offers a promising alternative. By allowing participants to adjust the intensity of each interval based on their perceived exertion, self-paced HIIT may be more tolerable and accessible for sedentary individuals, reducing barriers to exercise adherence (Buchheit and Laursen, 2013). This flexibility in intensity has the potential to improve exercise sustainability and outcomes, making it a viable option for real-world application, particularly for those with low baseline fitness levels (Kellogg et al., 2019; Allison et al., 2017).

Therefore the aims of the current study were: 1) to investigate the efficacy of self-paced SIT vs. LV-HIIT without encouragement on improving CRF and markers of cardiometabolic health; 2) to investigate the power output (PO) and heart rate (HR) responses to SIT and LV-HIIT within this environment; and 3) to explore whether the effort exerted within the protocols (in terms of PO and HR) influenced the magnitude of change in CRF. We hypothesised 6-week of LV-HIIT would result in superior improvements in CRF compared to SIT, as a result of greater compliance to exercise intensity. We also hypothesised participants producing the highest HR and PO responses within each intervention would improve their CRF to a greater extent than those with the lowest.

## Materials and methods

### Participants

Eighty-two previously sedentary males (n = 26) aged 18–45 and females (n = 56) aged 18–55, with a BMI ≤32 kg m^−2^, participated in the study (age 28 ± 10years, BMI 25 ± 3kg-m^−2^, VO_2peak_ 36.1 ± 7.6 mL kg^−1^ min^−1^). Participants were recruited via university wide email and posters displayed around the local community. Participant had a low risk of coronary heart disease (<10% in the next 5 years), assessed using the Framingham Heart Study Coronary Heart Disease Risk Prediction Score (Vasan et al., 2001). Participants undertaking >150 min of moderate-intensity exercise per week were excluded, assessed using the International Physical Activity Questionnaire. Pregnant or breast-feeding participants were also excluded. Participants gave written informed consent and all procedures were performed in compliance with the Declaration of Helsinki and were approved by the Coventry and Warwickshire NHS Research Ethics Committee.

### Study overview

The study used a randomised counterbalanced crossover design whereby participants completed two 6-week interventions: 1) SIT (30 s high-intensity self-paced cycling efforts interspersed with 120 s active recovery), and 2) LV-HIIT (1 min self-paced high-intensity cycling efforts interspersed with 1 min active recovery), separated by a 4-week wash out period.

Before each intervention (SIT and LV-HIIT) participants completed an incremental exercise test to exhaustion on an electromagnetically braked cycle ergometer to determine maximal aerobic power (Watt_max_ (W_max_)) and CRF (VO_2peak_), using an online gas collection system (MOXUS, AEI technologies, Pittsburgh, PA) as described previously (Scott et al., 2019). Briefly, the test consisted of 3-min stages starting at 25 W for females and 60 W for males. The workload was then increased by 35 W at each stage until participants could not maintain a cadence of >50 rpm or they reached volitional exhaustion. VO_2peak_ was taken as the highest value achieved during a 15 s recording period. HR was measured throughout the tests using a Polar H10 HR monitor (Kempele, Finland).

Seventy-two hours after the incremental exercise test participants returned to the laboratory following an overnight fast, having abstained from caffeine, alcohol and vigorous exercise the day before testing. Following 20 min of supine rest, blood pressure was measured in triplicate using a sphygmomanometer (Dinamap; GE Pro 300V2, Tampa, Florida). Aortic pulse wave velocity (aPWV) was then measured in triplicate using a SphygmoCor (AtCor Medical, Sydney, Australia) (Cocks et al., 2013). Body composition was analysed using Dual-energy X-ray Absorptiometry (Hologic QDR Series, Discovery A, Bedford, MA, United States). Finally, glucose tolerance was assessed using an oral glucose tolerance test (OGTT). A baseline 10 mL blood sample was taken before consumption of a 25% glucose beverage containing 75 g of glucose and 225 mL of water. Further 5 mL blood samples were collected 60 and 120 min after glucose ingestion. Blood samples were stored at −80°C until analysis. Plasma glucose concentrations were analysed using an automated analyser (Randox RX Series, the RX DaytonaTM).

### Post-intervention

Following both interventions, during the final week an incremental exercise test was performed instead of one of the scheduled exercise sessions. Seventy-2 hours after the final training session the post-training testing protocol was conducted with procedures, methods and timings identical in all respects to pre-training. During the four-week wash out period participants were instructed to return to their pre-intervention levels of physical activity.

### Training interventions

Participants trained 3 times per week (18 sessions in total), all training sessions were conducted in the laboratory at Liverpool John Moores University. As the aim of this trial was to assess the efficacy of self-paced SIT and LV-HIIT without encouragement, participants were withdrawn from the study if <90% of sessions were completed during each 6-week intervention, or if they did not attend at least 2 training sessions per week.

All training sessions were conducted on a Wattbike Trainer (Nottingham United Kingdom), which provides accurate PO data compared to the “gold standard” SRM Powermeter (Hopker et al., 2010). The use of the Wattbike allowed participants to manually adjust resistance using an airbrake, thereby controlling the exercise intensity by changing cadence or resistance. Participants were also provided with a HR monitor for all training sessions (Polar H10, Kempele, Finland). In keeping with the guidelines for HIIT (>80%HR_max_) (Roy, 2013), HR feedback was provided to allow participants to self-adjust their ‘effort’ in order to achieve a HR equivalent to >80% of their predicted HR maximum (PHR_max_, PHR_max_ = 220 – participants age). This equation is the most commonly used to calculate predicted maximal HR, and was used in the current study to enhance the real-world applicability, as it is not feasible to conduct maximal exercise testing within the general population. Only the HR data was made available to participants during sessions. The rest of the data recorded (cadence and PO) was hidden to replicate a real-world environment as this feedback is not visible on all commercially available cycle ergometers.

To further replicate a real-world environment, no encouragement was provided to participants during the training sessions. During the first session a single familiarisation interval was conducted, where the researcher encouraged the participant throughout the interval to help them achieve the appropriate HR response (>80% HR_max_). Following this, no further encouragement was provided, to imitate the conditions outside of a laboratory. However, to ensure the protocol was conducted correctly, the start and end of each interval and rest period was prompted by the researcher. Each training session began with a short warm up (5 min) of low intensity (self-paced) cycling.

### SIT

Participants performed repeated 30-s bouts of high-intensity effort interspersed with 120-s of active recovery (low-cadence cycling). Participants were instructed to reach >80% HR_max_ during each interval, and to achieve this either by a high cadence or high resistance, but due to the short interval duration an ‘all out’ approach was recommended. Participants completed 4 intervals per session in week 1, 5 in week 2, 6 in weeks 3–4, 7 in week 5 and finally 8 intervals per session in week 6. The total time commitment of each training session during week 6 was 20 min (Table 1).

**TABLE 1:** Characteristics of the SIT and LV-HIIT training programmes.

Week	SIT				LV-HIIT			
	Number of intervals	Total interval duration (min)	Total rest duration (min)	Total duration (min)	Number of intervals	Total interval duration (min)	Total rest duration (min)	Total duration (min)
1	4	2	8	10	6	6	6	12
2	5	2.5	10	12.5	7	7	7	14
3	6	3	12	15	8	8	8	16
4	6	3	12	15	8	8	8	16
5	7	3.5	14	17.5	9	9	9	18
6	8	4	16	20	10	10	10	20

### LV-HIIT

Participants performed repeated 1 min bouts of self-paced high-intensity effort interspersed with 1 min of active recovery (easy cycling). Participants were instructed to reach >80% HR_max_ during each interval, by either using a high cadence or high resistance, but due to the longer interval duration a durable cadence was suggested. Participants completed 6 intervals per session in week 1, 7 in week 2, 8 in weeks 3and4, 9 in week 5 and finally 10 intervals per session in week 6. The total time commitment of each training session at week 6 was 20 min (Table 1).

### Training session data analysis

During each training session the Wattbike PowerHub application (version 2.1.0) was used to record time, PO, cadence and HR. The lap counter function was used to mark the start and end of each interval. Following each training session, data was immediately downloaded to the Wattbike cloud-based storage application Wattbike Hub (https://hub.wattbike.com).

A peak HR (HR_peak_) and mean HR (HR_mean_) were determined for every interval, as was maximum PO (PO_peak_) and mean PO (PO_mean_) (Supplementary Figure S1). Average values for each training session were calculated, and then used to determine average values over the whole 6-week intervention (IHR_mean_, IPO_mean_, IHR_peak_, IPO_peak_). Only these mean values for the intervention are presented in the results section. All HR and PO data were normalised to the participants predicted HR_max_ (220-age) and W_max_ (calculated following the incremental exercise test), respectively. Data from the first training session where verbal encouragement was received was excluded from this analysis. Identical analysis methods were used for SIT and LV-HIIT.

Compliance to SIT and LV-HIIT was assessed based on previous intensity guidance for both HR and PO. HR compliance was the same for both SIT and HIIT (>80%HR_max_) in line with ACSM guidelines (Roy, 2013). The compliance for PO differed between SIT and LV-HIIT. Following from intensity guidance used in lab-based literature, PO intensity compliance was ≥200% W_max_ for SIT (Cocks et al., 2016) and ≥100% W_max_ for LV-HIIT (Little et al., 2010).

### Statistical analysis

The primary outcome was to compare the effect of time (Pre to Post) on CRF. The *a priori* power analysis indicated that 73 participants per intervention would be required to detect a 1 mL kg^−1^ min^−1^ difference in CRF(Clausen et al., 2018) with a power of 80% to detect an alpha of 0.05, assuming a standard deviation for the change in CRF of 3 mL kg^−1^ min^−1^ (Cocks et al., 2016; Scott et al., 2019) (G*Power Software Inc., Kiel, Germany). To account for a drop out of 20% the recruitment target was 88 participants. Previous work has suggested that a 1 mL kg^−1^ min^−1^ increase in CRF was associated with a clinically meaningful 10% reduction in cardiovascular mortality risk (Kavanagh et al., 2003). Measures taken pre- and post-training were analysed using a two-way within subjects ANOVA using the within subject factors time (Pre and Post) and intervention (SIT and LV-HIIT). Differences between the baseline and third visit and differences in HR and PO between training modes were analysed using a paired samples *t*-test. To evaluate the potential effect of exercise intensity on change in CRF participants were divided into 3 groups, and a one-way ANCOVA was completed, with baseline CRF as a covariate. The groups were based on exercise intensity, defined by IHR_peak_, IHR_mean_ or IPO_peak_, IPO_mean_. Participants were separated into tertiles (Low, Medium and High) for each of these variables (n = 27 in each group, Table 2 for characteristics). Plasma glucose responses were reported for 40 participants, as it was not possible to obtain blood samples from all participants. All analyses were performed using SPSS for windows version 26.0.0.1 (SPSS, Chicago, IL, United States), where an α–level of P < 0.05 was accepted as statistically significant. Data are presented as mean ± SD.

**TABLE 2:** Characteristics of groups based on exercise intensity.

	SIT		LV-HIT	
	Mean ± SD	Range	Mean ± SD	Range
IHR_peak_ (%HR_max_)				
Low	80 ± 3	69–82.9	81.2 ± 3.2	70.4–83.8
Medium	85.7 ± 1.6	83.0–87.9	86.1 ± 1.5	83.8–88.9
High	92.8 ± 2.8	88.0–99.9	93.5 ± 3.7	89.1–102.2
IHR_mean_ (%HR_max_)				
Low	70.7 ± 3.3	59.5–74.3	74.9 ± 3.2	62.9–77.9
Medium	77.8 ± 2.0	74.5–80.8	80.5 ± 1.8	78.2–83.6
High	85.5 ± 3.4	80.9–92.3	88.5 ± 3.7	83.6–96.3
IPO_peak_ (%W_max_)				
Low	192.6 ± 23.5	127.1–220.2	141.1 ± 11.9	106.3–154.6
Medium	237.7 ± 10.0	220.5–253.7	176.9 ± 11.8	161.2–193.7
High	302.0 ± 42.7	254.1–438.6	237.5 ± 41.0	196.2–326.2
IPO_mean_ (%W_max_)				
Low	132.9 ± 14.6	95.7–149.4	89.8 ± 9.3	63.0–98.0
Medium	159.3 ± 5.7	150.6–169.9	107.4 ± 4.6	98.2–113.5
High	187.1 ± 18.2	170.0–239.8	122.3 ± 8.7	114.0–145.3

IHR_peak_, interval peak heart rate as a percentage of predicted HR_max_; IHR_mean_, interval mean heart rate as a percentage of predicted HR_max_; IPO_peak_, peak power output as a percentage of W_max_; IPO_mean_, mean power output as a percentage of W_max_. N = 27 in all groups.

## Results

### Cardiorespiratory fitness and body composition

All CRF and body composition data and accompanying statistical outputs are presented in Table 3. No between intervention differences or interaction effects were detected in any of the variables relating to CRF or body composition (P > 0.05). Training improved CRF (main effect of time P < 0.001) and W_max_ (main effect of time P < 0.001). There was a significant main effect of time for reduction in whole-body absolute fat mass (*P* = 0.029) and body fat percentage (*P* = 0.02). No difference was observed over time for absolute lean mass (*P* = 0.853) or visceral fat mass (VAT) (*P =* 0.729).

**TABLE 3:** Cardiorespiratory fitness, body composition, cardiovascular-related outcomes and glucose tolerance pre and post SIT or LV-HIIT.

	SIT		LV-HIIT		*P* Value		
	Pre	Post	Pre	Post	Time	Intervention	Interaction
Cardiorespiratory Fitness							
VO_2peak_ (L.min^−1^)	2.5 ± 0.7	2.7 ± 0.7	2.5 ± 0.7	2.7 ± 0.8	*P =* 0.000*	*P =* 0.417	*P =* 0.718
VO_2peak_ (mL.kg^−1^.min^−1^)	36.3 ± 7.6	39.4 ± 8.0	36.1 ± 7.7	38.8 ± 8.9	*P =* 0.000*	*P =* 0.184	*P =* 0.406
Wattmax (W)	189 ± 52	215 ± 53	188 ± 49	212 ± 56	*P =* 0.000*	*P =* 0.458	*P =* 0.598
Body Composition							
Weight (kg)	69.7 ± 13.0	69.3 ± 12.7	69.8 ± 13.0	69.8 ± 12.9	*P =* 0.040*	*P =* 0.162	*P =* 0.499
BMI (kg-m^−2^)	24.8 ± 3.4	24.7 ± 3.3	24.8 ± 3.4	24.8 ± 3.4	*P =* 0.047*	*P =* 0.776	*P =* 0.268
Fat Mass (kg)	18.3 ± 6.4	18.1 ± 6.4	18.6 ± 6.5	18.0 ± 6.8	*P =* 0.029*	*P =* 0.675	*P =* 0.204
Lean Mass (kg)	45.9 ± 10.2	46.2 ± 10.0	46.2 ± 10.3	46.0 ± 11.0	*P =* 0.853	*P =* 0.921	*P =* 0.243
VAT Mass (g)	260 ± 149	262 ± 134	264 ± 156	266 ± 159	*P =* 0.729	*P =* 0.512	*P =* 0.995
Total Body Fat (%)	27.4 ± 7.1	27.1 ± 7.2	27.6 ± 7.2	27.2 ± 7.2	*P =* 0.002*	*P =* 0.276	*P =* 0.272
Cardiovascular Responses							
Blood Pressure (mmHg)							
Systolic	116 ± 10	115 ± 10	116 ± 11	114 ± 9	*P =* 0.018*	*P =* 0.254	*P =* 0.585
Diastolic	64 ± 6	64 ± 1	65 ± 7	63 ± 6	*P =* 0.054*	*P =* 0.573	*P =* 0.680
MAP	82 ± 6	81 ± 7	82 ± 7	80 ± 7	*P =* 0.014*	*P =* 0.411	*P =* 0.129
Resting Heart Rate (bpm)	66 ± 10	62 ± 10	64 ± 12	62 ± 9	*P =* 0.004*	*P =* 0.097	*P =* 0.971
PWV (m.s)	5.9 ± 1.1	5.7 ± 1.1	5.9 ± 1.1	5.7 ± 1.2	*P =* 0.002*	*P =* 0.953	*P =* 0.550
Glucose Tolerance							
Fasting Glucose (mmol.L^−1^)	4.4 ± 0.9	4.3 ± 1.0	4.5 ± 1.2	4.5 ± 0.8	*P =* 0.613	*P =* 0.328	*P =* 0.967
Glucose at 60 min (mmol.L^−1^)	5.9 ± 2.1	5.6 ± 1.0	5.6 ± 2.0	6.0 ± 2.2	*P =* 0.669	*P =* 0.459	*P =* 0.598
Glucose at 120 min (mmol.L^−1^)	5.0 ± 1.2	4.6 ± 1.6	4.6 ± 1.1	4.4 ± 1.6	*P =* 0.024*	*P =* 0.125	*P =* 0.422

Data reported as mean ± SD. *represents significant main effect for time (P < 0.05).

### Cardiometabolic responses

All cardiometabolic responses and accompanying statistical outputs are reported in Table 3. There were no significant between intervention differences or interaction effects for any of the cardiometabolic responses (*P* > 0.05, Table 3). Results revealed a significant main effect over time for systolic blood pressure (*P =* 0.018), mean arterial pressure (*P =* 0.014), resting HR (*P* = 0.004) and aPWV (*P* = 0.002). There was a significant main effect for glucose tolerance 120 min post OGTT (*P =* 0.024), where glucose at 120 min was significantly lower post intervention. No significant main effect over time was seen in diastolic blood pressure, fasting plasma glucose and glucose tolerance at 60 min post OGTT (*P >* 0.05, see Table 3).

### Effect of 4-week wash-out period

No variables measured following the wash-out period were different to baseline (P > 0.05) (Supplementary Table S1 for data and accompanying statistical outputs).

### Training intensity

**FIGURE 1:**
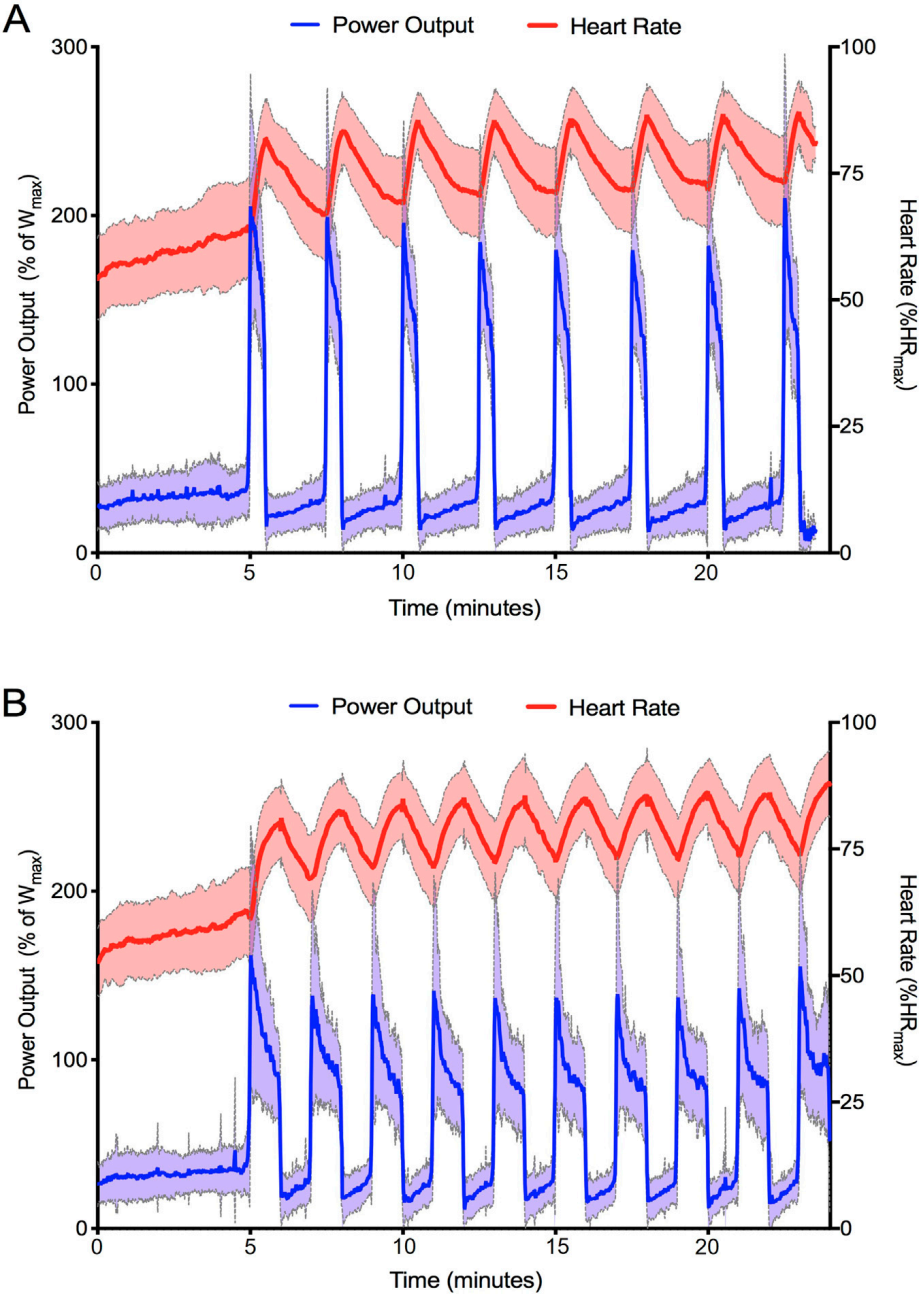
Average heart rate and power output achieved by all participants during the final training session of SIT **(A)** and LV-HIIT **(B)**. Data is presented as mean ± SD.

**FIGURE 2:**
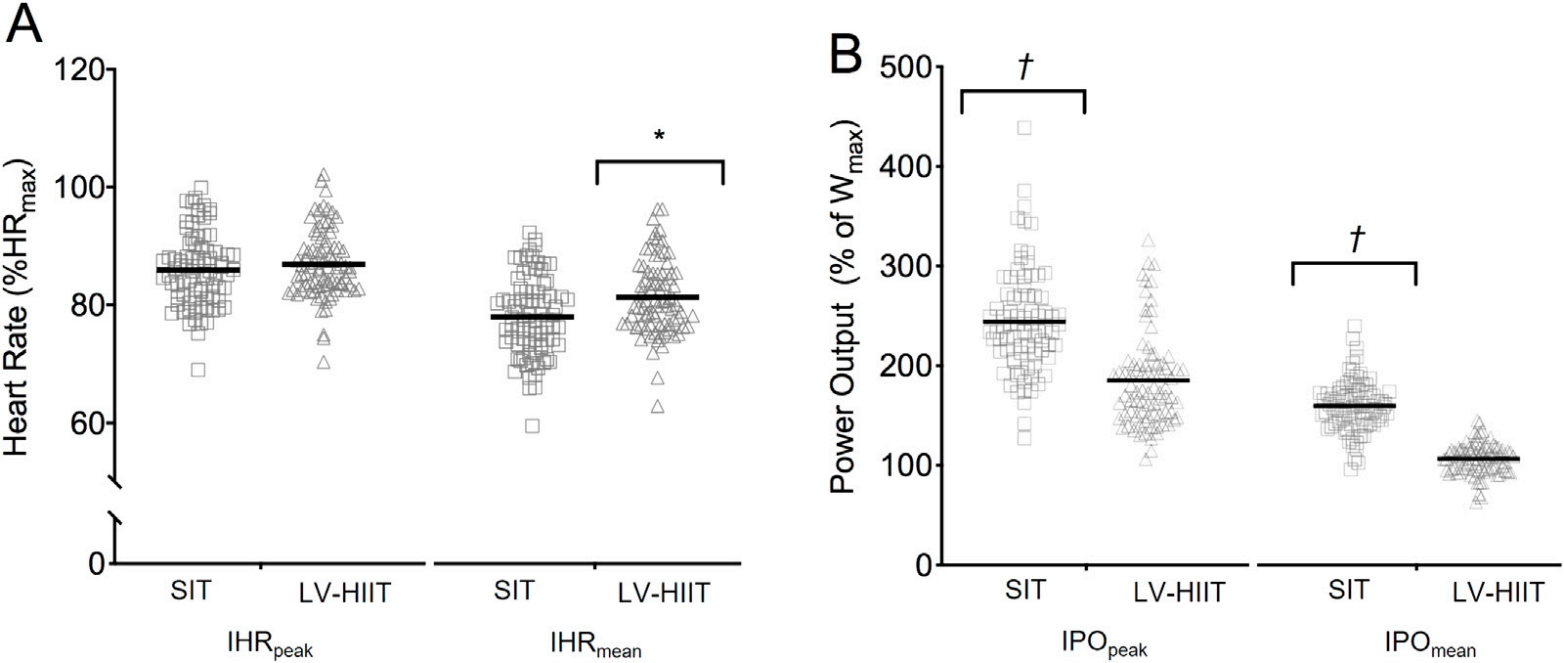
Average interval heart rate **(A)** and interval power output **(B)** achieved over the 6-week intervention. *indicates LV-HIIT significantly higher than SIT. + indicates SIT significantly higher than LV-HIIT.

Figure 1 shows the average HR and PO traces during the final recorded session of SIT (1A) and LV-HIIT (1B). SIT and LV-HIIT elicited similar IHR_peak_ (86% ± 6% vs. 87% ± 6% *P* = 0.327), whereas IHR_mean_ was greater during LV-HIIT (81% ± 7%) compared to SIT (80% ± 6%, *P* = 0.002). SIT elicited a higher IPO_peak_ than LV-HIIT (224 ± 53 W vs. 185 ± 47 W, P < 0.001), and a higher IPO_mean_ (SIT: 158 ± 26 W, LV-HIIT: 107 ± 15 W, *P* < 0.001). Figure 2 shows the individual responses to the interventions, and reveals the large range of IHR and IPO produced during SIT and LV-HIIT. HR and PO responses during each week of the interventions can be found in Table 4.

**TABLE 4:** Heart rate and power output responses during each week of training, for both SIT and LV-HIIT.

	Week 1	Week 2	Week 3	Week 4	Week 5	Week 6	P Value
IHR_peak_ (% HR_max_)							
SIT	88 ± 7	87 ± 7	86 ± 7	86 ± 6	85 ± 7	85 ± 7	*P <* 0.001
LV-HIIT	89 ± 6	88 ± 6	87 ± 6	86 ± 6	84 ± 13	85 ± 7	*P =* 0.003
IHR_mean_ (% HR_max_)							
SIT	81 ± 9	79 ± 8	80 ± 9	78 ± 7	77 ± 7	77 ± 8	*P <* 0.001
LV-HIIT	84 ± 6	82 ± 8	82 ± 7	81 ± 7	80 ± 8	79 ± 8	*P <* 0.001
IPO_peak_ (% W_max_)							
SIT	248 ± 56	244 ± 55	244 ± 54	248 ± 68	245 ± 60	245 ± 56	*P =* 0.760
LV-HIIT	184 ± 42	189 ± 45	188 ± 53	193 ± 60	189 ± 54	181 ± 59	*P =* 0.085
IPO_mean_ (%W_max_)							
SIT	165 ± 33	162 ± 37	160 ± 25	156 ± 27	158 ± 28	159 ± 26	*P =* 0.153
LV-HIIT	106 ± 17	109 ± 19	107 ± 16	108 ± 16	108 ± 19	105 ± 20	*P =* 0.230

IHR_peak_, interval peak heart rate as a percentage of predicted HR_max_; IHR_mean_, interval mean heart rate as a percentage of predicted HR_max_; IPO_peak_, peak power output as a percentage of W_max_; IPO_mean_, mean power output as a percentage of Wmax. Values are presented as Mean ± SD.

When considering the HR target 81% of participants achieved a IHR_peak_ >80%HR_max_ during SIT, and 94% during LV-HIIT. For IHR_mean_ this dropped to 41% (SIT) and 50% (LV-HIIT). When using PO as a measure of intensity, 65% of participants could achieve a IPO_peak_ ≥200% W_max_ during SIT (Cocks et al., 2016), and 100% achieved a IPO_peak_ ≥100%W_max_ during LV-HIIT (Little et al., 2010). When expressed as IPO_mean_ this reduced to 5% (SIT) and 63% (LV-HIIT).

### Exercise intensity and change in cardiorespiratory fitness

**FIGURE 3:**
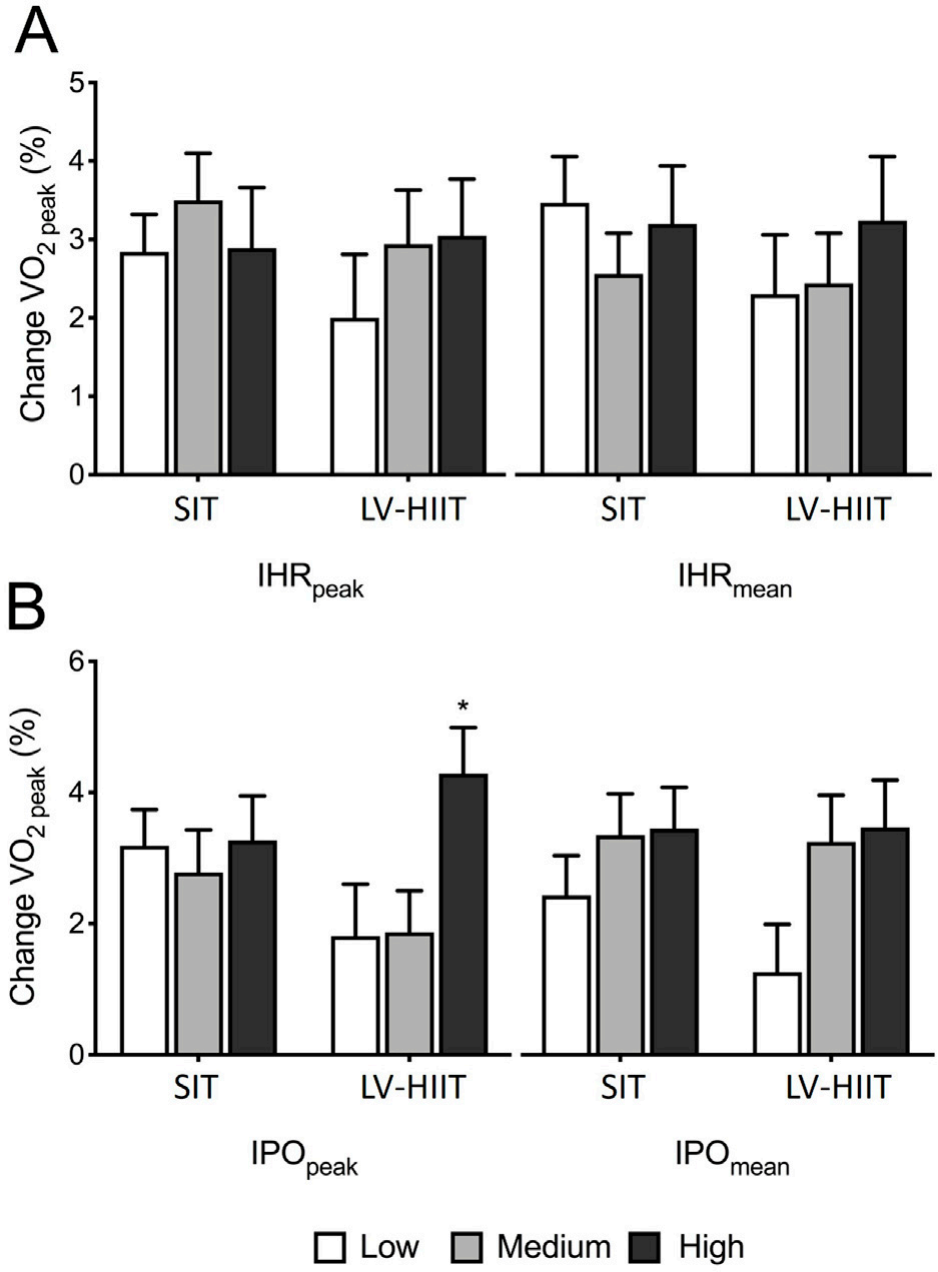
Relationship between exercise intensity and change in VO_2peak_ **(A)** Changes in VO_2peak_ categorised by heart rate peak (IHR_peak_) or heart rate mean (IHR_mean_). **(B)** Changes in VO_2peak_ categorised by peak power output (IPO_peak_) or mean power output (IPO_mean_). *indicates significantly higher than Low and Medium IPO_peak_ during LV-HIIT.

The relationship between exercise intensity (IHR and IPO) and change in VO_2peak_ is displayed in Figure 3. There were no significant differences in VO_2peak_ between participants who elicited a greater IHR_peak_ or IHR_mean_ compared to those with a lower IHR (Figure 3A), in both SIT (*P =* 0.677 IHR_peak_, *P =* 0.535 IHR_mean_) and LV-HIIT (*P =* 0.549 IHR_peak_, *P =* 0.617 IHR_mean_).

In LV-HIIT, participants in the High IPO_peak_ group had a significantly greater change in VO_2peak_ compared participants producing a Low or Medium IPO_peak_ (Low +1.8 ± 4.1 Medium +1.8 ± 3.2 and High +4.2 ± 3.6 mL kg^−1^⋅min^−1^, *P =* 0.020). Change in VO_2peak_ was not significantly different between participants producing a Low, Medium or High IPO_peak_ during SIT (Low 3.2 ± 2.8, Medium 2.8 ± 3.4 and High 3.3 ± 3.5 mL kg^−1^ min^−1^, *P =* 0.886) (Figure 3B). In LV-HIIT, there was a trend (*P =* 0.074) towards participants with a Medium and High IPO_mean,_ having a greater change in VO_2peak_ compared to the Low group (Low: +1.3 ± 3.8 mL kg^−1^ min^−1^, Medium: +3.3 ± 3.7 mL kg^−1^ min^−1^, High: +3.5 ± 3.7 mL kg^−1^ min^−1^). There was no significant difference in VO_2peak_ when grouped based on IPO_mean_ during SIT (*P =* 0.398) (Figure 3B).

## Discussion

This was the first study to employ a randomised cross-over design to investigate SIT and LV-HIIT protocols using readily available gym equipment, without provision of verbal encouragement. The most important finding of the present study is that 6-week of SIT or LV-HIIT resulted in similar improvements in CRF and cardiometabolic health. Both SIT and LV-HIIT elicited high compliance to the prescribed HR target of >80%HR_max_ despite being self-paced and performed without encouragement. Despite all participants being given the same intensity target (>80%HR_max_ during the intervals), a large range of individual HR and PO responses were observed within the protocols. Interestingly, similar increases in CRF were observed regardless of HR achieved during SIT or LV-HIIT. However, greater increases in CRF were observed in participants who produced a higher IPO_peak_ but only during LV-HIIT.

### Comparison of cardiometabolic health responses to SIT and LV-HIT

SIT and LV-HIIT have been advertised as an efficacious and time-efficient exercise mode for improving cardiometabolic health (Batacan et al., 2017; Campbell et al., 2019). Interestingly, despite large differences between protocols, as seen in the HR and PO profiles (Figure 3), 6-week of self-paced SIT or LV-HIIT resulted in similar positive adaptations to cardio-metabolic health.

In contrast to our hypothesis SIT and LV-HIIT produced similar improvements in CRF in the current trial, despite being performed without encouragement and at self-paced intensities. This is in line with previous meta-analyses (Rosenblat et al., 2020; de Oliveira-Nunes et al., 2021) conducted primarily using laboratory based interventions using target exercise intensities and strong verbal encouragement. This finding has importance given that CRF is strong predictor of mortality (Blair et al., 1989), and improvements in CRF are associated with a reduction in all-cause mortality (Blair et al., 1995). Previous work has suggested that a 1 mL kg^−1^ min^−1^ increase in CRF was associated with a 10% reduction in cardiovascular mortality risk (Kavanagh et al., 2003) and a 45-day increase in longevity (Clausen et al., 2018). Improvements in CRF following SIT or LV-HIIT may be partially attributed to increased mitochondrial biogenesis through the upregulation of peroxisome proliferator-activated receptor γ coactivator (PGC)-1α. This master regulator of mitochondrial adaptations is activated by the high muscle fibre recruitment and cellular stress experienced during intense intervals, triggering signalling cascades (e.g., AMPK, p38 MAPK, CaMK) that enhance oxidative capacity and cardiometabolic health (Little et al., 2011; Gibala et al., 2009). In addition, recent evidence from a systematic review and meta-analysis suggests that improvements in CRF are primarily driven by central cardiovascular adaptations, such as increased stroke volume and cardiac output, with minimal contribution from haematological changes (Astorino et al., 2022). Therefore, either SIT or LV-HIIT could be prescribed within an unsupervised gym environment with the aim of increasing CRF and reducing mortality.

Additionally, results from the current study showed modest but significant changes in body composition with a decrease in body fat % following SIT (−0.3%) or LV-HIIT (0.4%). This is similar to the results seen in a recent meta-analysis comparing HIIT to non-exercise controls (Steele et al., 2021), however >5% of weight loss is a generally accepted criterion for clinically meaningful weight loss within the literature (Horn et al., 2022). Both SIT and LV-HIT resulted in a decrease in MAP (SIT: 2 mmHg; LV-HIT: 2 mmHg) and resting HR (SIT: 3bpm; LV-HIIT: -2bpm). Although these results were not clinically significant (MAP >10 mmHg) (Ettehad et al., 2016), improvements were similar to a previous gym-based study using a varied LV-HIIT protocol (15–60s intervals) (Shepherd et al., 2015). Finally, the current study found small improvements in glucose response during a 2 h OGTT, regardless of intervention (−0.4 mmol.L-1 following SIT and −0.2 mmol.L-1 following LV-HIIT). It has been suggested that the 2 h response following an OGTT is a stronger predictor of mortality than fasting glucose (DECODE Study Group, 2003; Metter et al., 2008). Taken together these results suggest that when completed outside of a laboratory environment without constant encouragement SIT or LV-HIIT can be successful at improving cardio-metabolic health. Although some caution should be applied as improvements to body composition, blood pressure and glucose tolerance failed to reach clinical significance.

### Effect of intensity on cardiorespiratory fitness

This is the first study to investigate the effect of intensity on CRF using self-paced SIT or LV-HIIT. Surprisingly, the current research found similar improvements in CRF regardless of the peak or mean HR achieved during intervals in either SIT or LV-HIIT. In patients with coronary heart disease, Moholdt et al. (2014) demonstrated that there was a dose-response relationship even when participants performed interval exercise in the 85–95%HR_max_ intensity zone. Following 12-week of aerobic interval training (4 × 4 minute intervals, with 3 min rest) a greater improvement in CRF_k_ (∼2 mL kg^−1^ min^−1^) was observed when patients HR exceeded 92%HR_max_, compared to <92%HR_max_. This conflicting result could be explained by the interval duration, as the protocol used by Moholdt et al. (2014) consisted of longer intervals (4 min). Additionally, it has previously been suggested that HR achieved during intervals may not be suitable for prescription of SIT and LV-HIIT, as HR lag at exercise onset results in a slower response to exercise intensity compared to a VO_2_ response (Cerretelli and Di Prampero, 1971; Buchheit and Laursen, 2013). Therefore, using IHR_peak_ or IHR_mean_ to analyse LV-HIIT intervals (<60s) may not be appropriate, and so the use of alternative analysis methods, e.g., time spent above 80%HR_max_, and its effect on CRF should be investigated in SIT and LV-HIIT.

Although HR did not predict changes in CRF, HR is still a useful and accessible prescription tool for sedentary populations. In the current study, the prescribed HR (>80%HR_max_) was achieved by the majority of participants during each interval, and this intensity was enough to elicit clinically significant improvements in CRF (∼2 mL kg^−1^ min^−1^) even when participants were classified in the lowest intensity HR tertile. This has important practical implications as public health researchers have previously questioned whether sedentary participants would be able to achieve the required intensity to elicit health benefits when exercise was performed without encouragement (Hardcastle et al., 2014; Biddle and Batterham, 2015).

This study was the first to use a readily available gym bike which allowed collection of PO data during self-paced HIIT (Wattbike), therefore individual PO could also be used to assess the impact of exercise intensity on CRF. The results suggest participants producing a High IPO_peak_ during LV-HIIT induced greater CRF advances compared to their Low or Medium counterparts. However, surprisingly, changes in CRF failed to reach significance when grouped based upon IPO_mean_. One possible explanation for this is that although participants in the current study achieved similar IPO_mean_ compared to previous lab-based research (100%W_max_), it has to be noted that the ‘power profile’ for LV-HIIT differed from earlier studies (Little et al., 2010). Previous research has traditionally used a constant load approach during 1 min intervals, whereby the same power is applied throughout based on %VO_2peak_ or %HR_max_. However, in the current study, with the freedom to perform the intervals using self-pacing, our participants’ power profile during LV-HIIT was similar to that seen in a Wingate (with a large initial peak in PO), rather than mimicking the constant load approach. Importantly, although significant differences in CRF existed between the IPO_peak_ tertiles for LV-HIIT, participants in all groups achieved clinically significant improvements in CRF(Clausen et al., 2018) (Low: + 1.8 mL kg^−1^ min^−1^, Medium: + 1.9 mL kg^−1^ min^−1^ High: + 2.3 mL kg^−1^ min^−1^).

In contrast to LV-HIIT, it appears there was no relationship between changes in CRF and PO intensity during SIT, despite large variation in PO responses (IPO_peak_: Low 193%, Medium 237%, High 302% W_max_). It has been established that intensity of exercise is important for many key physiological adaptations, and that an increasing exercise intensity can mediate responses to training, e.g., CRF and mitochondrial adaptations (MacInnis and Gibala, 2017). Therefore, when studying SIT protocols, the methods used in the current study (HR and PO) may not be encompassing the changes in exercise intensity that impact adaptations to exercise. A recent study Fiorenza et al. (2018) found the PGC-1α mRNA response to SIT (18 × 5 s “all-out” sprints with 30s rest vs. 6 × 20 s “all-out” sprints) was greater when exercise induced the highest muscle lactate accumulation, the greatest drop in muscle pH, and the highest plasma adrenaline levels. Therefore, in order to improve prescription, future research should investigate further the effects of exercise intensity on health outcomes such as CRF and utilise these alternative intensity measurements.

### Limitations

There are some limitations that should be considered when interpreting the results and designing future interventions. Firstly, sedentary but otherwise healthy participants were recruited, therefore we cannot generalise these results to populations with cardiovascular or metabolic disease. Second, due to the large number of participants completing the intervention simultaneously and resource constraints, we were unable to assess participants’ habitual physical activity levels and diet throughout the study period. However, participants were instructed to maintain their habitual diet and physical activity levels for the duration of the study. Additionally, data on menopausal status were not collected, which may have influenced some of the outcomes assessed. The use of a shorter incremental protocol with more aggressive power output increments may have resulted in lower PPO values compared to traditional ramp protocols, potentially affecting the intensity of the prescribed training. Finally, this was a relatively short intervention (6-week), therefore we do not know the long-term effects of self-paced SIT or LV-HIIT or how intensity may influence outcomes over a longer duration.

### Conclusion

In summary, this study demonstrates that 6-week of self-paced SIT or LV-HIIT result in similar improvements to CRF even when performed without encouragement. This suggests that either SIT or LV-HIIT can be successfully prescribed to a sedentary or at-risk population to improve cardiometabolic health. Additionally, this study found similar increases in CRF regardless of HR achieved during the intervals. Interestingly, larger increases in CRF were observed in participants who elicited a higher IPO_peak_ but only in LV-HIIT. Importantly, even participants in the Low HR or PO categories showed significant improvements in CRF, suggesting a HR target of >80%HR_max_ is an effective intensity prescription for HIIT that stimulates positive health adaptations.

## Data Availability

The raw data supporting the conclusions of this article will be made available by the authors, without undue reservation.
