# Supplementary material for: Effect of self-paced sprint interval training and low-volume HIIT on cardiorespiratory fitness: the role of heart rate and power output
Source: Front Physiol. 2025 Feb 5;16:1484722. doi: 10.3389/fphys.2025.1484722 (PMC11835828; doi:10.3389/fphys.2025.1484722)
Supplement: Supplementary file 1 [file Table1.docx]

|  | Time Point | | *P* Value |
| --- | --- | --- | --- |
|  | Baseline 1 | Baseline 2 | Time |
| Cardiorespiratory Fitness |  |  |  |
| VO_2peak_ (l.min^-1^) | 2.5 ± 0.7 | 2.5 ± 0.7 | *P=*0.893 |
| VO_2peak_ (ml.kg^-1^.min^-1^) | 36.1 ± 7.6 | 36.3 ± 7.6 | *P=*0.839 |
| Wattmax (W) | 186 ± 51 | 190 ± 51 | *P=*0.0 |
| Body Composition |  |  |  |
| Weight (kg) | 69.9 ± 13.1 | 69.9 ± 12.9 | *P=*0.877 |
| BMI (kg-m^-2^) | 24.8 ± 3.4 | 24.7 ± 24.7 | *P=*0.930 |
| Fat Mass (kg) | 18.3 ± 6.2 | 18.5 ± 6.5 | *P=*0.795 |
| Lean Mass (kg) | 46.1 ± 1.0 | 46.0 ± 1.0 | *P=*0.939 |
| VAT Mass (g) | 258 ± 154 | 263 ± 150 | *P=*0.861 |
| Total Body Fat (%) | 27.6 ± 7.6 | 27.8 ± 7.6 | *P=*0.864 |
| Cardiovascular Responses |  |  |  |
| Blood Pressure (mmHg) |  |  |  |
| *Systolic* | 116 ± 10 | 115 ± 10 | *P=*0.556 |
| *Diastolic* | 65 ± 7 | 64 ± 6 | *P=0.217* |
| *MAP* | 82 ± 7 | 81 ± 7 | *P=*0.306 |
| Resting Heart Rate (bpm) | 65 ± 11 | 66 ± 10 | *P=*0.691 |
| PWV (m.s) | 6.0 ± 1.1 | 5.9 ± 1.0 | *P=*0.401 |
| Glucose Tolerance |  |  |  |
| Fasting Glucose (mmol.L^-1^) | 4.6 ± 1.5 | 4.3 ± 1.0 | *P=*0.090 |
| Glucose at 60min (mmol.L^-1^) | 5.9 ± 2.2 | 5.6 ± 2.0 | *P=*0.512 |
| Glucose at 120min (mmol.L^-1^) | 4.7 ± 1.1 | 4.6 ± 0.9 | *P=*0.396 |

**Supplementary Table 1.** Cardiorespiratory fitness, body composition, cardiovascular-related outcomes and glucose tolerance at baseline visit 1 and baseline visit 2.
