# Supplementary figures and images for: Effect of self-paced sprint interval training and low-volume HIIT on cardiorespiratory fitness: the role of heart rate and power output
Source: Front Physiol. 2025 Feb 5;16:1484722. doi: 10.3389/fphys.2025.1484722 (PMC11835828; doi:10.3389/fphys.2025.1484722)

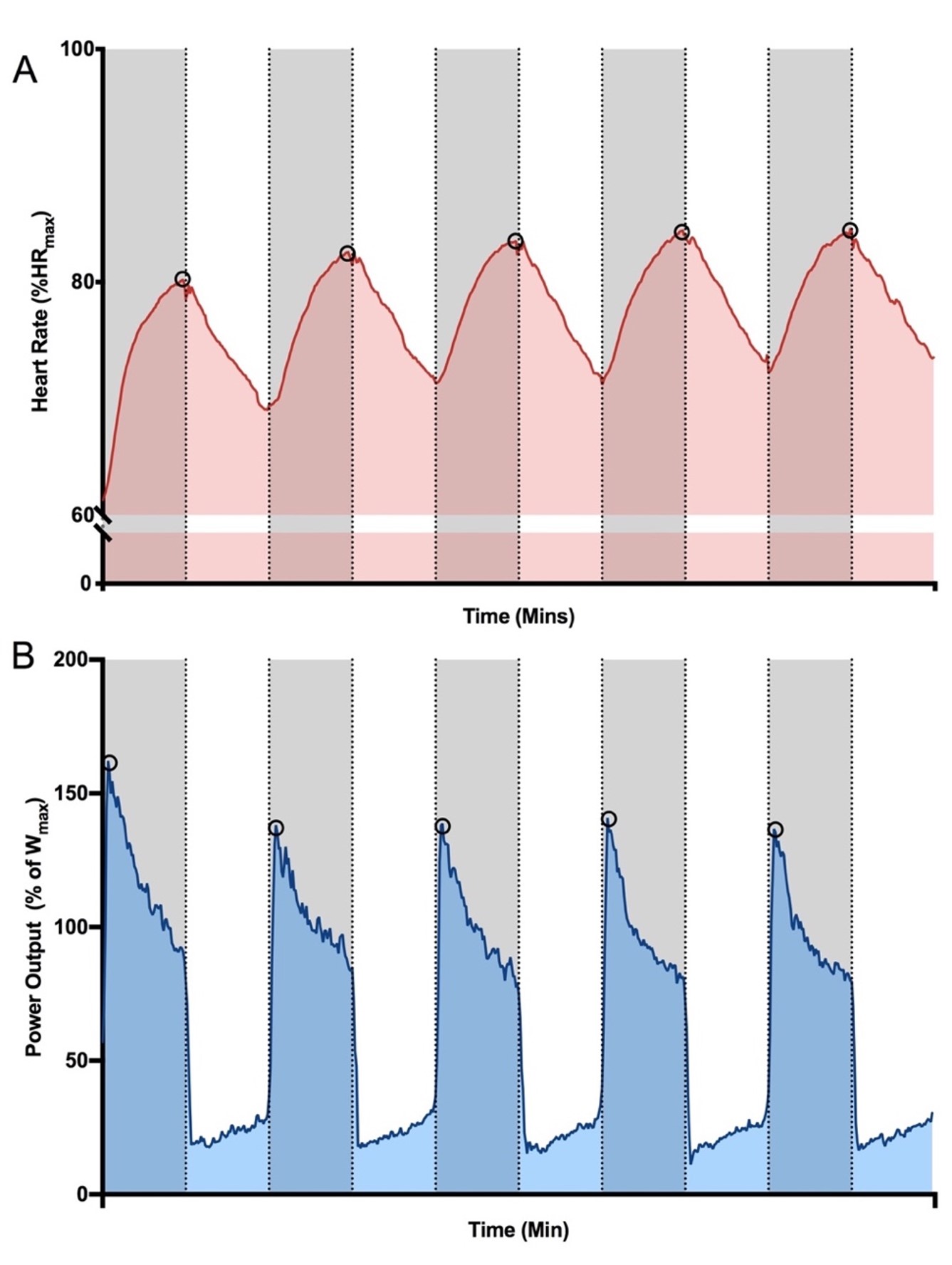

Supplement: Supplementary file 2 [file Image1.jpg]
